# Supplementary material for: Comparison of static and dynamic cerebral autoregulation under anesthesia influence in a controlled animal model
Source: PLoS One. 2021 Jan 8;16(1):e0245291. doi: 10.1371/journal.pone.0245291 (PMC7794034; doi:10.1371/journal.pone.0245291)
Supplement: Attachment — Submitted filename: Response to Reviewers.pdf [file pone.0245291.s001.pdf]

Response to reviewers

We like to thank the reviewers and editors for their valuable comments and suggestions to this manuscript. We have made an effort to answer all questions and implement suggestions to the best of our ability. Our response to the reviewers questions are printed in blue letters, where additions to the revised manuscript are colored in green. Quotes from the old manuscripts are colored in red. The original question remains in black letters. We took the liberty to numerate the comments for cross-referencing purposes.

Reviewer #1: There are several areas which should be clarified by the authors:

1. No data for arterial O2 and CO2 are provided. This is key as CO2 is a powerful regulator of CBF. Given the length of the preparations (16.2 h), it is probable that atelectasis to some degree occurred due to prolonged supine placement. Data for O2 and CO2 MUST be provided to validate the conclusions drawn.

The reviewer is raising a valid concern which we have previously failed to acknowledge in the manuscript, but that we have now addressed in this revision. In brief, we maintained end tidal carbon dioxide (ETCO2) within a small range (typically ~30 mm Hg) throughout all of the experiments, and monitored peak inspiratory pressure. In some cases we would block the outflow of a respiration partially in order to ensure full lung expansion on the next breath. While we did observe some variation in ETCO2 in our experiments, our results are more consistent with fluctuations in ETCO2 caused by the changes in heart rate rather than causally influencing the intracranial pressure given the small range. More detail is provided below.

Supplemental O2 given during the ventilator based respiration was kept constant. Pulsatile arterial oxygenation (SpO2) was measured and used for adequate ventilation control in conjunction with CO2. CO2 is known to regulate cerebral blood flow, as the reviewer has correctly emphasized. Based on the reviewer's suggestion, we performed additional analysis of the data and found that partial pressure of ETCO2 and heart rate (HR) in our experiments were correlated, while HR was correlated with arterial blood pressure (ABP). We expect that the small fluctuations in ETCO2 would likely be caused by the animal's reaction to intracranial pressure (ICP) manipulations from our experiment. A typical reaction to increased ICP is an increase in ABP, often realized by an increase in HR itself, which might in return influence ETCO2 measurements. A clarifying plot showing the change in ETCO2 as compared to pressure measurements and HR as well as the fixed respiration rate (RR) is given below.

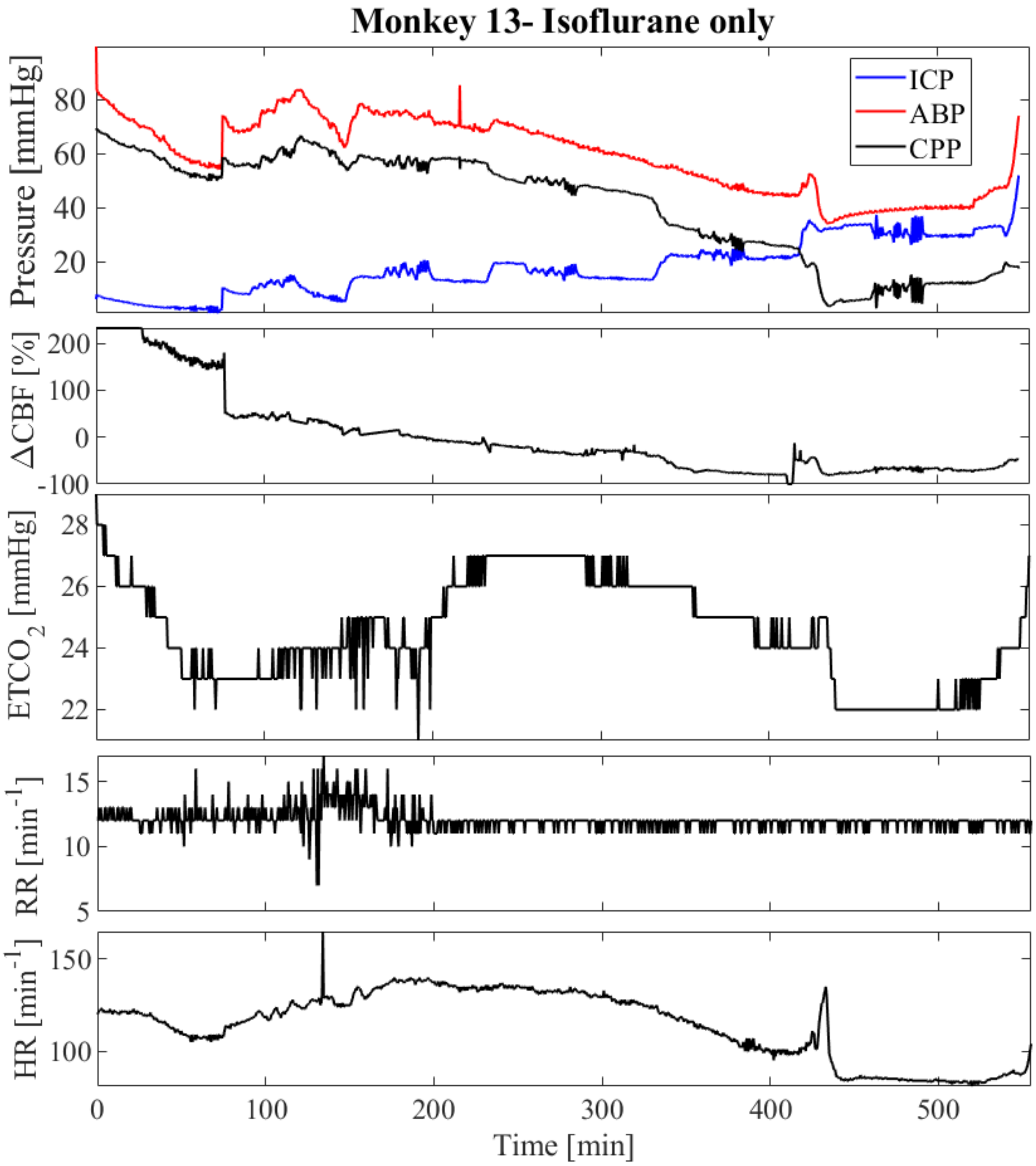
Monkey 13- Isoflurane only
Pressure [mmHg]
ICP
ABP
CPP
ΔCBF [%]
ETCO2 [mmHg]
RR [min-1]
HR [min-1]
Time [min]

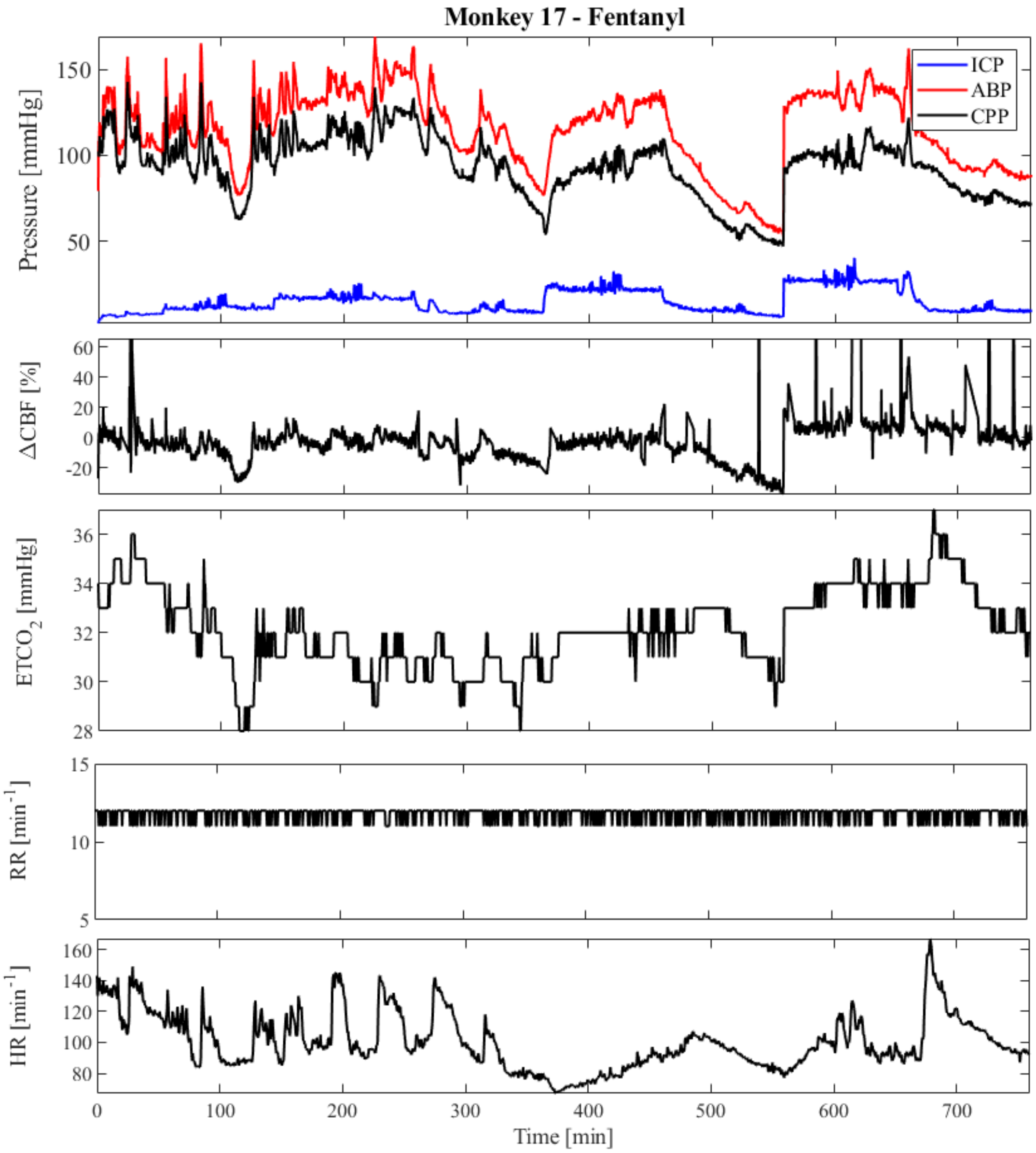

Our hypothesis is strengthened by largely stable ETCO2 values, only fluctuating in a small range around a normal average. The figure below shows the standard deviation and mean of all animals used in this study for the ETCO2 readings, averaged over the entire experiment.

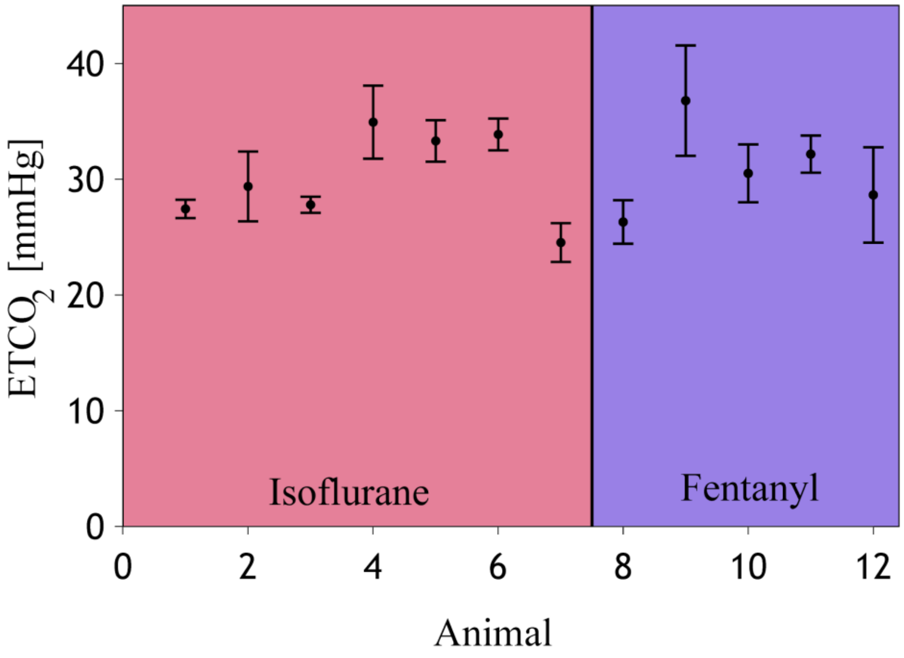

For non-human primates, when not under anesthesia, a normal range of ETCO2 in generally understood to be between 35 and 45 mmHg, while under anesthesia without assistive ventilation substantial larger values may be observed as outlined in multiple non-human primate anesthesia guidelines (https://www.researchservices.umn.edu/services-name/research-animal-resources/research-support/guidelines/anesthesia-non-human-primates, https://research.utexas.edu/wp-content/uploads/sites/7/2020/02/NHP_Anesthesia_guidance_ARC_010920.pdf). We therefore conclude that CO2 levels were not directly harming the animals' ability to autoregulate and perfuse the brain. Because of the small range and no evidence of ETCO2-driven ABP events, we assume that CO2 variation does not change our conclusions.

We have added the graph of averages and standard deviations in the revised manuscript as figure 3 on page 11. We have made sure to explain our measurement devices in the methods section by adding "To monitor sufficient anesthesia, heart rate, respiration rate, pulsatile arterial blood oxygenation (SpO2), end tidal carbon dioxide partial pressure (ETCO2), and other vital signs were recorded at 2 samples per minute through the SurgiVet veterinary monitor (Smiths Medical, Minneapolis, MN, USA). " and have added information about the ETCO2 changes in the results in the form of the figure above. We added the figure caption: "**Partial pressure in end tidal CO_2 averages per animal.** The error bar shows the standard deviation around the mean $ETCO_2$ calculated across all measurements of each individual animal. The left side highlighted in red shows isoflurane anesthetized animals, the right side shows predominantly fentanyl anesthetized subjects." and pointed towards these results on page 11 in lines 8 and 9: "The ETCO2 value calculated as an average of the entire duration of the experiment on individual animals was showing no values outside an expected range [35,36], indicating appropriate anesthesia (Fig. 3).". For the discussion, we added the following sentences when discussing the Lassen's curve and the influence of CPP on CBF: "CO2 is a known regulator of CBF and ABP. While we did see a positive correlation between ABP and ETCO2, our ETCO2

values were confined within a small range within normal limits (Fig 3). We therefore assume that ETCO2 was not the leading cause of CBF changes, but rather a consequence of increased heart rate and ABP as an autoregulatory response to induced ICP changes.”.

2. The animals were paralyzed. Why? CO2 level will vary as depth of paralysis varies. How was such depth of paralysis monitored? Again, it is imperative to provide data for CO2 to properly interpret and confirm conclusions regarding effects on CA determined in this study. IACUC often forbids use of paralysis without clear strong proper justification.

The reviewer is raising a good point. All of our experiments, including the use of paralytic, were approved by the IACUC of the University of Pittsburgh, where the experiments were performed. In an effort to maximize the usefulness and knowledge gain of this terminal experiment, several measurements were run in parallel and some of these involved testing visual responses as measured with electroencephalography (EEG). Paralysis of the animals was required for measurements of visual evoked potentials in an effort to maintain the direction of gaze of the animal, which otherwise drifts during anesthesia. ETCO2 levels were observed and measured throughout the experiment, along with heart rate, SPO2, and arterial blood pressure, as indicators for sufficient anesthesia. Paralysis depth was confirmed by performing a toe pinch test, monitoring the EEG, and looking for sudden changes in HR or ABP which, in order to evaluate if the animal was not properly anesthetized. This was not observed in any measurement. To emphasize this in the manuscript, we have added the following information to the experimental setup section: “The paralytic was necessary for a parallel experiment we ran in the same subjects. The infusion of paralytic at a constant dose was not expected to interfere with the autoregulatory assessment. ”. The information added in response to question 1 will also serve to address this concern.

3. This paper appears a bit improperly framed. It really is a study of anesthetics and their effects on CA. In particular, the authors appear quietly to advocate use of anesthetics to test CA intactness instead of other typical techniques (thigh cuff) for which they feel there is unnecessary pain/risk. Perhaps, therefore, the title of the MS should be altered to reflect this agenda and the corresponding study hypothesis re-articulated to emphasize that this is a study of anesthetics and CA. If so, this paper really should be published in an anesthesia journal.

The reviewer’s observation has been taken into consideration and we have changed the title to: “Comparison of static and dynamic cerebral autoregulation under anesthesia influence in a controlled animal model”. To clarify our aim of advocating anesthesia as an effective model for comparison of the performance of different CA measurement techniques, we changed the abstract slightly. The sentence “. Given oscillation and baseline changes in blood pressure and intracranial pressure, we conclude that cerebral perfusion pressure is the most likely descriptor of cerebral autoregulation impairment. ” appeared redundant and was removed. In its place we extended the statement about anesthesia to: “Static and dynamic CA both show impairment in high dose isoflurane anesthesia, while low isoflurane in combination with fentanyl anesthesia maintains CA, offering a repeatable animal model for CA studies.”.

While we understand that this observational work is focused on anesthesia and its role in cerebral autoregulation, we believe that the interested reader group is broader than found in a journal focused on anesthesia. Therefore, we believe that PloSOne is the right platform for this type of study.

4. Going along with the above, the authors do not provide proper documentation that many others have studied the effects of anesthetics on CA. The authors should provide an enhanced discussion on this topic. If properly framed, then, what is really new here? Certainly, the experiments were conducted rigorously with newer techniques for CA determination (DCS), but truly the idea that anesthetics affect CA is not at all new. This paper appears to only incrementally add to the fund of knowledge in the literature.

We would like to emphasize that improvements we made over previous studies include not only a more rigorous study design in which we tested both ICP and ABP influences on CA, but also the advanced measurement of microvascular blood flow through DCS. In addition, we are comparing static and dynamic autoregulation while also offering information on non-evoked dynamic measurements, here called pseudo-dynamic CA. Therefore, this work offers a larger variety of methods and a wider range of perturbations to the cerebrovascular system. Furthermore, although we do not agree that our work only incrementally adds to the literature, the review criteria for PLoS One explicitly do not include novelty or importance as among the criteria for acceptance (https://journals.plos.org/plosone/s/reviewer-guidelines#loc-what-to-assess).

We acknowledge that we could have better compared our work with other studies in regards to the choice of anesthesia. We therefore included the following paragraph in the discussion: "Our observation of impaired autoregulation under isoflurane anesthesia in static and dynamic CA is shared with early findings from Strebel et al. [35]. They measured the impairment of dynamic CA through thigh cuff occlusion, finding the rate or regulation in transcranial Doppler measurements of the middle cerebral artery to be reduced. However, they report that static CA is only marginally changed, indicating that it is still intact, which differs from our observations. Another study by Summors et al. [36] demonstrated a severe loss in the rate of regulation, measured in the same way as Strebel et al., reporting a loss of dynamic CA under isoflurane. Our approach differed from these findings by firstly by measuring the microvasculature through DCS, rather than relying on cascading reactions to the major arteries. Secondly, we compared the influence of ABP and ICP independently under the conditions of different anesthetics. The two different pressure challenges have shown to differ in their response, while reaching similar conclusions (see Fig 6)." citing (Strebel S, Lam AM, Matta B, Mayberg TS, Aaslid R, Newell DW. Dynamic and Static Cerebral Autoregulation during Isoflurane, Desflurane, and Propofol Anesthesia. Anesthesiology. 1995;83: 66–76.) and (Summors AC, Gupta AK, Matta BF. Dynamic cerebral autoregulation during sevoflurane anesthesia: A comparison with isoflurane. Anesth Analg. 1999;88: 341–345.).

5. What is the power for these studies? The n is quite small (7 and 5) for the 2 experimental groups. Only males were used with no justification. Therefore, these studies are not at all

consistent with present emphasis on Rigor. In particular, this is important since LLA and CA may be dependent on gender.

While the reviewer is right about potential differences in sex and, compared to clinical trials, a low number of subjects, we like to emphasize that these studies are performed on non-human primates. These animals were part of other research activities before they were used in this terminal study. This use of animals for multiple experimental purposes is in alignment with the three Rs of animal research (Replacement, Reduction and Refinement) to reduce the amount of animals used in research and maximize the use of every individual animal to the scientific community. For this reason, we were not able to control the sex of the animals. Furthermore, we like to point out that the power in these studies for statistical analysis is not necessarily derived from the number of animals but rather from the length of data collection and the number of repeated measurements of the same pattern of ICP and ABP oscillations. This testing of statistical significance in multiple measurements from individual animals is consistent with a very substantial body of literature in non-human primates, which are a scarce experimental resource. To make this limitation clear, we have added the following sentences to the experimental design section of the methods chapter: "All animals have been part of previous scientific experiments, before they were used in this study, following the guidelines to reduce the number of animals in scientific experiments to a minimum and maximize their use. Due to these limitations, we were not able to influence the age and sex distribution of the subject pool and can therefore not assess these variables in our study. ".

Reviewer #2: The manuscript was described well except for the following points. Please rewrite these points properly.

1. The paragraph starting with; "Static Autoregulation" (P8L23-P9L8), is difficult to understand how to observe data. Please rephrase the whole paragraph with clear explanations for the following terms and phrases; "beta-value", "Laser instabilities", "intensity auto-correlation at zero-delay time", "beta started fluctuating" and "alpha Db value".

The reviewer has raised a valid concern about the clarity of this paragraph and we have taken action to rephrase it as follows: "The CBF data was cleaned from laser instabilities and artifacts by z-score rejection ($z > 0.5$) on an individual NHP basis. Laser instabilities were identified based on the β value, defined as the y-axis intersection of the measured speckle pattern intensity auto-correlation in DCS, subtracted by 1. The fluctuations of the β value are expected to be very small and any larger deviation can be discarded as non-physiological noise. When laser stability was lost, presumably due to internal reflections of light at the laser to fiber interface, β started fluctuating significantly and that time point was identified as unstable by its z-score. A measurement was said to have artifacts if at any time point the β value was smaller than the median (approx. 0.1 for larger fiber core diameters and 0.5 for single mode fibers) by 0.01. CBF data were cleaned further by rejecting any averaged 10 second window of CBF outside a z-score range of 2 standard deviations, rejecting further artifacts from room light, motion, and laser instability not removed previously." We would like to point out that these technical details

are in reference to the new blood flow measurement technique DCS, which encountered technical difficulties during some of the measurements that have since been fixed. The paragraph follows after the description of this new technique in the methods section of this manuscript where all used terms are explained and further literature references are offered.

2. It is difficult to understand a new term of "Pseudo-Dynamic" autoregulation. The concept for this newly defined phrase must be explained.

We understand that the term is new and may be confusing. We therefore were careful to describe the phrase pseudo-dynamic autoregulation in the introduction (Page 4, Lines 17-21) where it reads: "This method has been used to determine the optimal CPP (CPP_{opt}), which is where PRx is the smallest, in order to optimize CA intactness and patient outcome [22]. Because the calculation of CPP_{opt} shows a range of CA intactness, the otherwise dynamic PRx assessment receives static CA characteristics, which is why it is here referred to as a *pseudo-dynamic CA*.". The idea behind this terminology is to distinguish methods that can either give a CPP range of intact CA (static) and methods that show a degree of impairment at any given time (dynamic). The PRx value can provide both. It is a metric of CBF as a function of CPP, but also time. This unique hybrid type of CA measurements, able to provide both static and dynamic information, is thus referred to as pseudo-dynamic. To improve the clarity of this statement, we have rephrased it to: "The calculation of CPP_{opt} shows a range of CA intactness, which is a static CA characteristic. At the same time, the dynamic PRx assessment allows to determine a degree of CA impairment. Therefore, PRx is a hybrid method and here referred to as a *pseudo-dynamic CA*. ".

Most legends for the Figures are insufficient to understand contents well because of poor definition or poor drawing of x-axis and y-axis.

1. Fig.1: Need explanation in the legend how to control and measure airway pressure for and how to this is transferred to MAP, and also how to control saline reservoir sinusoid way.

MAP was manipulated through active control of the ventilator, and is described in the Methods and the figure caption. The ventilator used in this experiment can be connected to a PC via USB cable and commands to change the PEEP value can be sent regularly to mimic a sinusoidal pattern. The description of active programming is therefore an accurate description and we do not believe that any additional description would lead to additional information. The circular motion of the ICP, achieved by rotating a reservoir of saline on a lever arm around a center point was added to the figure caption for clarification. The description of Figure 1b) now reads "The saline reservoir connected to the ventricle catheter was raised and lowered in a circular motion on a lever, whose center point was placed relative to the NHP's head to allow for ICP manipulation."

2. Fig.2: Need explanation for zoom-up on the right, how to obtain sinusoidal curves of the ICP. It is unclear why the ABP follows the ICP because the ABP must be independent of ICP. Describe how the CPP was measured.

The zoomed in window, shows a close up of an ICP oscillation followed by an ABP oscillation in one example experiment, and this is explained in the figure legend. The sinusoidal curves were

obtained by oscillation of the saline reservoir in a circular motion by an electric motor, therefore inducing more or less fluid pressure, dependent on the lever position, which changes ICP periodically. We have modified Fig 1's caption above to make that point more clear.

The impression that ICP is independent of ABP is only true in the case in which ICP is changed and ABP is not able to respond. For intact autoregulation, we expected and found that slow changes in ICP are followed by a change in ABP, which regulates the CPP and maintains adequate blood flow. Given the various mechanisms in which CA operates, an absolute independence of ABP and ICP seems unlikely. The term "pressure reactivity" has been established in the field of cerebral autoregulation and was described by Fraser et al., who used the phase lag between ABP and ICP to successfully quantify CA impairment. We have adopted said mechanism in this article.

Cerebral perfusion pressure is the pressure gradient between arteries leading into the brain and veins leading out of it. The inflowing pressure is approximated with systemic arterial blood pressure measured in the carotid artery. While the venous pressure can not be easily measured directly, it is clinical standard to approximate it with intracranial pressure. Therefore, the cerebral perfusion pressure in this paper is, in accordance to clinical standard, calculated as the difference of mean arterial pressure and intracranial pressure. We added the following sentence to the figure caption to make this approximation apparent: "CPP is defined as the difference between MAP and ICP. ".

3. Fig.3: Need explanation for histograms of CPP, ABP and ICP. In the bottom graph, need explanation how to calculate the measurement for each isoflurane percentage.

We are unclear about the point the reviewer is making. The histograms show the number of data points across all animals given their anesthesia categories, that fell into the respective bin of 1 mmHg. The graphs are thus labeled as "#Data Points" and "CPP/ABP/ICP [mmHg]". The last histogram regarding the Isoflurane percentage used is showing the number of measurements, not datapoints. A measurement consists of a set of ICP oscillations followed by a set of ABP oscillations at a given ICP baseline value. Because the ISO% was only recorded every 15 minutes on a manual anesthesia protocol, the sampling rate is not sufficient to create a comparable graph to the histograms above. Furthermore, the ISO% remains unchanged for long periods of time, often unchanged for the entire day consisting of multiple measurements, that any other representation would be misleading. We have added information about the bin size and the nature of the number of measurements for isoflurane percentages to the figure caption:

"Here the red line indicates isoflurane anesthesia and the blue line represents the group with mainly fentanyl anesthesia. Shaded areas show the standard deviation across all NHPs in this group. The histograms are based on group averaged data for CPP, ABP, and ICP (from the top), with histogram bin sizes of 1 mmHg. A Mann-Whitney U-test was performed to test statistical significance between fentanyl and isoflurane groups. The bottom graph shows the distribution of isoflurane percentage for the anesthetic groups with the number of measurements (of approx. one-hour length each). Isoflurane percentages were recorded manually every 15

minutes, and an average across one recording (approx. 90 minutes of unchanged ICP baseline) was calculated."

4. Fig.4: Do not hide the representative line for isoflurane under the fentanyl SD curves.

We would like to thank the reviewer for pointing us towards this detail. We have revised the figure and brought the representative line to the foreground again.

In addition, several terminologies were not defined properly. Need clear definition for the following terms in main text.

1. PRx (pressure reactivity index) (P4L11)

Assuming that the reviewer unfortunately missed the description given on page 4 in lines 8 to 11, we like to highlight that the pressure reactivity index was described as follows: "Pressure reactivity measurements are another method to describe autoregulation intactness and was introduced by Czosnyka et al. [21], where a moving Pearson correlation between ICP and ABP during naturally occurring changes of pressures within a 5 minute window describes pressure reactivity, known as the pressure reactivity index (PRx). " We have added the information that a 5 minute time window was used for this Pearson correlation calculation, to add additional detail.

2. PEEP (positive end-expiratory pressure) oscillation (P5L5)

To address this shortcoming we have added the following clarifying sentence on page 4 after line 5: "PEEP describes the positive pressure that is maintained inside the trachea and lungs after exhalation in positive pressure respiration ventilators. "

3. alphaDb (diffuse coefficient) (P6L6)

In the document the term was described as "This term describes the diffusion of red blood cells in the brain and has previously been linked to and correlated with cerebral blood flow [32–34]. " We then referenced papers for more information. To provide more detail in our manuscript, we have added the following explanation: "The α term describes the ratio of moving scattering particles (red blood cells) to static particles (such as blood vessels, brain and muscle tissue at rest). D_b describes the Brownian motion diffusion coefficient, which is a direct derivative of the Brownian motion model used to derive the solution to the diffusion equation for scattering light in tissue. Because α is largely constant, its influence on the rate of blood flow change is negligible, such that αD_b can be treated as one variable in the fitting algorithm."
