# Supplementary material for: Comparison of static and dynamic cerebral autoregulation under anesthesia influence in a controlled animal model
Source: PLoS One. 2021 Jan 8;16(1):e0245291. doi: 10.1371/journal.pone.0245291 (PMC7794034; doi:10.1371/journal.pone.0245291)

Response to the reviewers

We like to thank the editor and reviewers once again for their service and consideration of this manuscript. We have previously answered all questions and implemented suggestions to the best of our abilities and believe that the remaining request of reviewer #2 is based on a misunderstanding of the nature of our Figure 4. Below is our response to the reviewers.

Reviewer #1: The authors have provided appropriate address of prior comments and have made suitable revisions in the new MS.

We like to thank reviewer 1 for the valuable contribution to this manuscript.

Reviewer #2: The MS revised almost properly except for the comment on Fig.4. This reviewer requested to clearly plot average lines for two anesthesia, Isoflurane and Fentanyl. However, Fig.4 has not been revised in the revised MS. Please revise Fig.4 as this reviewer requested, because average lines are important to evaluate the static autoregulation.

Otherwise, MS is properly revised.

First, we like to also thank reviewer 2 for the valuable contribution to the manuscript which has truly benefited the clarity of this paper. Unfortunately, we had difficulties understanding the request raised by the reviewer above.

In our initial response we assumed the reviewer was referring to the red line being behind the blue shaded area, making it appear purple colored in the figure. This design has been revised, such that the two average lines now lay on top of the two shaded areas and no color change occurs. If this was not the reviewer's intention, we have misunderstood the request and like to apologize for this confusion.

Now we think that the reviewer might have been misled to believe that the red curve ends because it is hidden behind the shaded area denoting the standard deviation of the blue curve in the top graph of Figure 4 of the manuscript (see figure below at approx. 100 mmHg). We like to point out that the shaded areas are transparent and would show the line of averages if we had data for isoflurane at such high cerebral perfusion pressure. This is made clear by plotting the histogram of cerebral perfusion pressure underneath the Lassen's curve. Here one can see that the CPP values for the red distribution (Isoflurane-only group) are clearly missing at these higher values and can therefore not be shown in the Lassen's curve above. Therefore, all average lines are shown as intended, and no further revision is needed.

If this is not the information the reviewer was asking for, we would like to ask the reviewer or editor to further clarify the point made such that we can improve this graphic as intended.

Figure 4 of Revision 1

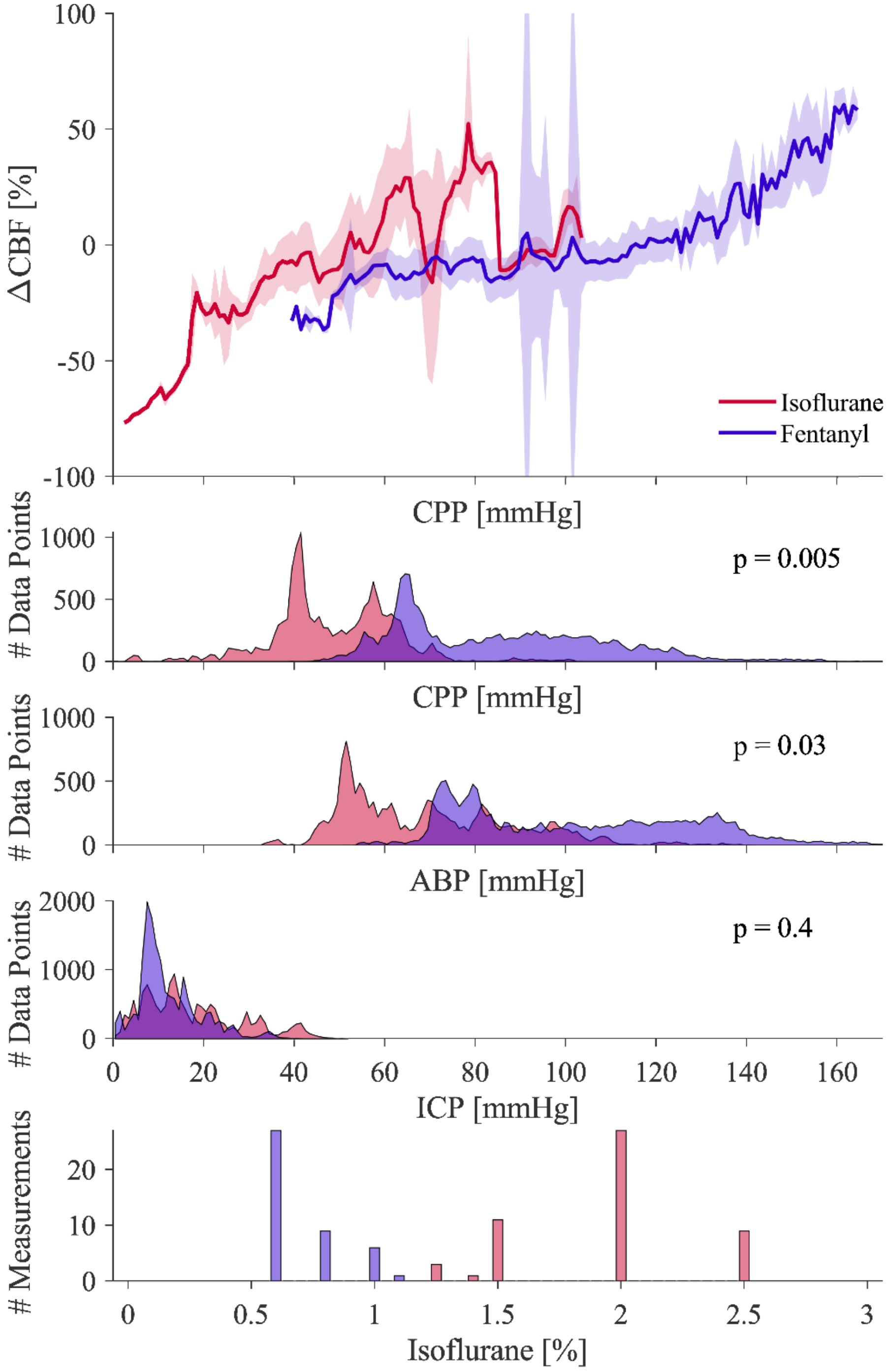

Supplement: Attachment — Submitted filename: Response to Reviewers.pdf [file pone.0245291.s002.pdf]
